# Incidental parasitic infestations in surgically removed appendices: a retrospective analysis

**DOI:** 10.1186/1746-1596-2-16

**Published:** 2007-05-24

**Authors:** Özgür Aydin

**Affiliations:** 1 Department of Pathology, Alanya Hospital, Baskent University, Antalya, Turkey; 2 Saray Mah. Yunusemre Cad. No:1 07400 Alanya Antalya Turkey

## Abstract

**Background:**

Appendiceal parasites can cause symptoms of appendiceal pain, independent of microscopic evidence of acute inflammation. The diagnosis of a parasitic infestation is generally achieved only after the pathologic examination of the resected appendices.

**Patients/Methods:**

Pathology department records were reviewed for all patients who required an operation for symptoms of acute appendicitis between 2000 and 2006. The specimens which were pathologically diagnosed to contain parasites were reevaluated for features of acute inflamation, and parasite type. The medical records were reviewed in detail to achieve a diagnostic score(Eskelinen). Radiologic imaging findings were correlated, if present.

**Results:**

Of the 190 appendectomies performed, 6 specimens (3,15%) were found to contain parasites(4 Enterobius vermicularis, 2 Taenia subspecies). Appendectomies with Taenia showed acute inflamation, while acute inflamation was absent in the ones with Enterobius vermicularis. The Eskelinen score was higher than the treshold in two cases with an acute inflamation, and in two without. Ultrasound scans, and a computed tomography scan were performed in 5 patients. In 3 of 4 bland appendices, results favored acute appendicitis.

**Conclusion:**

The diagnosis of gastrointestinal parasites is not only made by examining the stool but the diagnosis can be made by histology from surgical specimens. Timely diagnosis and appropriate therapy might prevent probable future complications that may necessitate surgical procedures, at least in some of the patients. The clinical management of these infections is different from that for classical appendicitis.

## Background

Suspected acute appendicitis is the most frequent cause for emergency operations in visceral surgery, worldwide. The lifetime risk of acute appendicitis for men and women is 8.6% and 6.7%, respectively, however, the lifetime risk of having an appendectomy is 12% for men and 25% for women [1]. A reported 250,000 appendectomies are performed annually, with approximately 15% of these resulting in the removal of normal appendices [2]. A history of migratory pain together with physical findings and leukocytosis is generally percieved as accurate diagnostic clues for children and adults. The overwhelming majority of parasitic infestations of appendix are not associated with an acute inflamation[3]; thus considered to be a component of false acute appendicitis.

Interestingly, the presence of parasites in the appendix may cause an appendiceal colic even without eliciting an acute inflamation. The appendiceal colic due to a parasitic infestation is explained by the hypothesis of appendiceal lumen obstruction. Laboratory findings of these patiens show a great variability including extremely high to normal values. Radiologic imaging features of the issue is not well described in the literature. Patients may have multiple previous visits to hospitals due to abdominal discomfort, but unfortunatelly missing the diagnosis leads to the inevitable surgical operation. Our study presents a subgroup of patients with clinical, laboratory, and radiologic findings, that might have avoided surgery, if diagnosed previously.

## Methods

Pathology department records were reviewed for all patients who required an operation for symptoms of acute appendicitis between July 2000 to July 2006. The haematoxylin and eosin stained sections, which had been prepared from resected specimens of these 6 cases were reevaluated and classified due to presence of acute inflamation and parasite type. In cases without acute inflamation whole appendix was examined in order not to miss a focal lesion.

The medical records of these 6 patients were reviewed in detail. It was seen that all patients were presented to at least 2 doctors, one being the referred surgeon. Calculation of the Eskelinen diagnostic scores and interpretation of the results were performed according to published methods[4]. A cut-off value of 55 score was considered in favor of acute appendicitis. The previously described Eskelinen score (table 1) was preferred because the clinical parameters of the Eskelinen score system were nearly routinely recorded, and easily reached. Ultrasound scans, and a computed tomography scan were performed in 5 patients.

**Table 1:** Clinical parameters and their weights by the Eskelinen score

**Symptom/sign**	**Criterion, points**	**Factor**
Tenderness	2 = RLQ, 1 = any other location	11.41
Rigidity	2 = yes, 1 = no	6.62
Leucocyte count	2 = ≥ 10,000 G/l, 1 = <10,000 G/l	5.88
Rebound tenderness	2 = yes, 1 = no	4.25
Pain at presentation	2 = RLQ, 1 = any other location	3.51
Duration of pain	2 = <48 hours, 1 = ≥ 48 hours	2.13

RLQ: right lower quadrant

Negative appendectomy is defined as one which is performed for a clinical diagnosis of acute appendicitis but in which the appendix is found to be normal on histopathological examination.

## Results

190 appendectomies were performed with the preoperative diagnosis of acute appendicitis at The Baskent University Alanya Hospital from 2000 through 2006. Of the 190 appendectomies performed, 6 specimens (3,15%) were found to contain parasites at pathological examinations.

**Figure 1:**
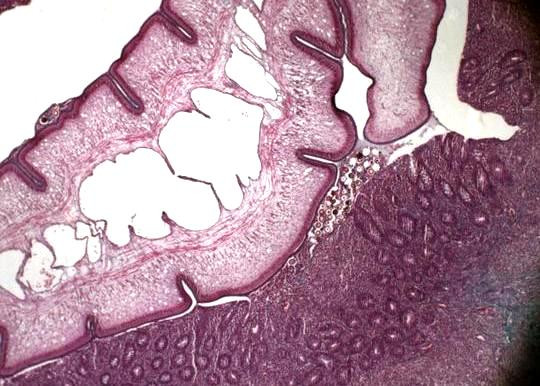
the fragment of helminthe was seen in the lumina as an elongated and flattened segment with eggs(original magnification × 40).

**Figure 2:**
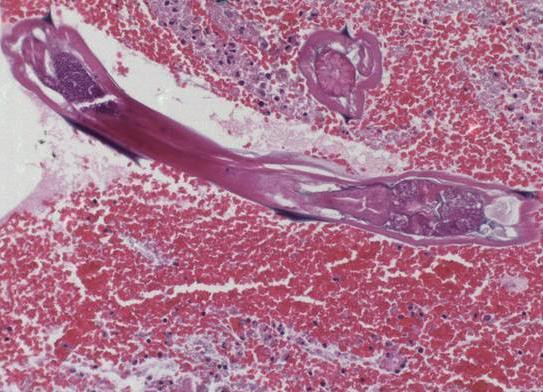
one cross, and one trans section of E. vermicularis with characteristic hooks at both sides(original magnification × 40).

In 4 of cases, the parasites were identified as Enterobius vermicularis(E. vermicularis); and in 2 as Taenia subspecies(Taenia spp.). In cases with Taenia, appendices showed the macroscopic and microscopic features of acute appendicitis. A formalin fixed segment of the parasite had been observed, but not recognised by the technician who had handled the specimen. Microscopic slides presented mucosal ulceration, and luminal exudate accompanied by an elongated and flattened segment of the helminthe. A large number of round eggs with a thick radially striated shell were within the parasites uterus and were also freely floating in the lumina(Fig. 1). The characteristics allowed to conclude that the helminthe belonged to the genus Taenia. In cases with E. vermicularis, macroscopic examination showed bright and swollen appendices with serosal congestion. Histopathology revealed the lack of acute inflamation, only after many serial sections. Appendices contained luminal vegetable material and many transverse or vertical transected pinworms with a chronic inflammatory infiltrate predominated by eosinophils(Fig 2).

Patients were 3 males and 3 females. The ages ranged from 8 to 30 years. The medical records of these 6 patients showed that, the ones showing a Taeniasis infection underwent an appendicectomy at their first presentation in the hospital. Other 4, had at least 1 more previous visit with a complaint of abdominal discomfort. One of them was even followed with a suspicion of acute appendicitis, but a surgery was not performed as the clinical signs receded in follow up. As retrospectively calculated, prior to surgery, the 2 of the patients with acute appendicitis had an Eskelinen score higher than 55. The 2 of the patients with a normal appendix had also an Eskelinen score higher than 55. 1 of the remaining patients with a normal appendix had an obviously low score, while the other had a close score to the threshold (table 2). Abdominal ultrasound scans was performed in 4, and a computed tomography scan in 1 patient while 1 patient did not have any. Ultrasound scans favored an acute appendicitis in 1 of the 2 acute appendicitis; diagnosed 2 of normal appendices as acute appendicitis; and was congruent with acute appendicitis in 1 of the normal appendix cases. 1 patient with a histologically normal appendix was evaluated as "suspicius for acute appendicitis" in computed tomography scan. The patient profile and results are listed in Table 3.

**Table 2:** Eskelinen scoring of patients according to findings.

Patient no	Tenderness	Rigidity	Leucocyte count	Rebound tenderness	Pain at presentation	Duration of pain
1	2	1	18,8	1	2	2
2	2	1	10,5	2	2	2
3	1	1	4,73	1	1	1
4	1	2	14,2	2	1	2
5	2	2	11,9	2	2	1
6	2	2	14,5	2	2	2

**Table 3:** The patient profile and results.

Patient no	Age	Gender	Eskelinen score	USG/CT results	Acute inflamation	Parasite
1	8	female	56,73	AA	negative	E. vermicularis
2	8	female	60,98	AA	negative	E. vermicularis
3	10	male	33,80	NA	negative	E. vermicularis
4	27	male	52,68	AA?	negative	E. vermicularis
5	25	male	65,47		positive	Taenia spp.
6	30	female	67,06	AA	positive	Taenia spp.

USG: ultrasound scans, CT: computed tomography, AA: acute appendicitis, NA: normal appendix

## Discussion

Parasitosis is a public health problem in endemic countries with temperate climates. A variety in geography is noted[5-8]. While often considered tropical, parasitic diseases are now seen more frequently in developed countries because of immigration and increased world travel. Intraluminal parasites within the resected appendix specimen is generally an incidental finding. The role of parasites in acute appendicitis is discussed [3]. They mostly accompany a noninflamed appendix. In retrospective studies they constitute only a minor percentage of false acute appendicitis.

Gastrointestinal infection due to Enterobius vermicularis occurs worldwide and is considered to be the most common helminth infection. Although seen in all ages and socioeconomic levels, there is a distinct predilection for children and youngsters. Pinworms are usually asymptomatic inhabitants of the intestine. In children who exhibit intense pruritus in the perianal region, which may be associated with symptoms like loss of appetite, insomnia and restlessness, pinworm infection should be suspected. Diagnosis may be achieved by direct visualization of the adult worms or microscopic detection of eggs in a fecal flotation, but only a minority of patients have eggs in their stool. A night-time application of cellophane tape in the perianal area can serve as an easy way to manage the diagnosis. The parasite wanders widely inside the bowel including the appendix. Worldwide, the reported incidence of Enterobius infestation in patients with symptoms of appendicitis ranges from 0.2–41.8%[9]. The association of Enterobius infestation and appendicitis was first described in 1899[10]. Since then, there have been several studies describing this entity [9,11,12].

The simple presence of E. vermicularis in the appendix can produce symptoms of acute appendicitis[9]. E. vermicularis infestation of the appendix can produce clinical features of acute appendicitis, referred to as 'appendiceal colic', independent of histological acute inflammation. Instead, either no tissue reaction or a chronic inflammatory infiltrate of eosinophils is associated.

Taeniasis is a well-known worm infection, characterized by the presence of the helminth in the human intestine. Infection occurs frequently in individuals who eat undercooked beef or pork. Most cases of infection do not cause any symptoms, while others may produce abdominal pain, weight loss, digestive disturbances. Infection is generally recognized when the segments of the parasite appear in the stool or exit through the anus. The occurrence of Taenia spp. in the cecal appendix is so rare that the situation invites case reports[13].

Parasites can definitely be associated with the evolution of classic appendicitis. In series, there exists a range of pathologic findings from nonspecific changes to frankly ruptured appendicitis[9]. Observations show that their presence may indicate an luminal obstruction. Ova release from female parasites may be a feature of appendiceal obstruction, which consequently is followed by bacterial overgrowth and finally ending to acute appendicitis. The reversibility of the process may be questioned. One of the patints in our study, had a clinical history of a privious appendiceal pain, but no surgery was performed as he got well in observation.

An appendiceal colic caused by parasitic infestation can not be differenciated from the right lower quadrant pain of usual acute appendicitis. Clinical and laboratory findings of an infection are generally observed as the intestinal system is already involved by the parasites. In some cases a careful history may point to antecedent symptoms and a time course that are incompatible with typical appendicitis, but physical examination is generally not specific enough to differentiate between parasitic and ordinary appendiceal pain. Blood work for eosinophilia and a rapid examination of the stools by an experienced technician may serve some help. When suspected, these patients may benefit from clinical observation and re-evaluation before proceeding directly to emergency appendectomy. If such patients are not improving after a period of observation, further diagnostic studies are recommended. Radiologic procedures are strongly experience dependent, and these cases may easily be missed as seen in our study. The surgeon, handling a patient with a right lower quadrant pain, and a radiology suspecting an acute appenciditis, may face a noninflamed appendix in operation. The appendix hosting the parasites without an acute inflamation will put the surgeon in trouble, as excision of every appendix is questioned after the more common use of laparoscopy. Surgery should be planned with attension especially in children and youth age groups, travelars, and immigrants.

Appendectomy, open or laparoscopic, should proceed with caution if the appendix is observed not to be acutely inflamed. The surgeon must bare in mind the possibility of resident worms in the vermiform appendix. The application of laparoscopic appendectomy technique in patients with parasitic infestation requires some technical considerations[14]. It may be troublesome to deal with worms released after the appendix has been divided, especially if the surgeon is not prepared for it.

It is imperative that patients receive antihelminthic treatment afterwords, because the appendectomy treats only a consequence but not the root of the disease. E. vermicularis infestation is treated with an oral dose of mebendazole, which is repeated in 1–2 weeks. Reinfection may be expected, because humans do not develop a protective immunity against pinworms. In cases of Taeniasis, spesific species identification is not required for treatment and patients are treated with a single dose of praziquantel.

## Conclusion

The present report describes a group of patients who had experienced a curable infectious disease, but unfortunately had undergone a surgery with potential complications. Patients with intestinal parasitic infections generally have previous visits to hospitals with abdominal discomfort problems, before the clinical features pose an acute abdomen that will lead to the emergent operation. Only a high index of suspicion, and particularly taking parasitic origin in concern in differential diagnosis of abdominal disturbances might hopefully prevent a surgery. Pathologically, the surgical material should be examined with the fecaloid material, as the eggs or the parasite itself may be within. The surgeon should be aware that the clinical management of these cases is different from that for an ordinary appendicitis, as it requires antihelminthic treatment.
